# Evaluation of Perceived Pain, Discomfort, Functional Impairments, and Satisfaction When Relieving Crowded Lower Anterior Teeth in Young Adult Patients Using Corticision-Assisted Fixed Orthodontic Treatment: A Randomized Controlled Trial

**DOI:** 10.7759/cureus.26489

**Published:** 2022-07-01

**Authors:** Mohamad Radwan Sirri, Ahmad S Burhan, Mohammad Y Hajeer, Fehmieh R Nawaya

**Affiliations:** 1 Department of Orthodontics, University of Damascus Faculty of Dentistry, Damascus, SYR; 2 Department of Pediatric Dentistry, Syrian Private University Faculty of Dentistry, Damascus, SYR

**Keywords:** swelling, mastication, satisfaction, discomfort, pain, surgical blade, mild and moderate crowding, visual analog scale, corticision

## Abstract

Background

No previous trial (randomized and controlled) studied the levels of pain, discomfort, and acceptability associated with acceleration of dental movement during orthodontic treatment using corticision. The purpose of this study is to compare the pain, discomfort, ease of procedure, patient satisfaction, and analgesic use during corticision-assisted (without extraction) decrowding of the lower anterior teeth with the traditional orthodontic method.

Materials and Methods

Fifty-two patients (38 females, 14 males; mean age: 21.38 ± 1.05) were randomly distributed into two groups: the corticision group (CORT, n=26) and the control group (CONT, n=26). Corticision was applied by a surgical blade and a hammer at three anterior regions on the lower jaw using three radiological guides. The levels of pain, discomfort, swelling and chewing difficulties were registered on a visual analog scale (VAS) at one, seven, and 14 days after applying the first wire (0.14-inch NiTi archwire). Questionnaires were administered to assess the level of satisfaction, ease of the procedure, and the number of analgesics patients took. Mann-Whitney U tests were used to detect significant differences between the two groups. The Chi-Square test was used to study the significance of differences in taking analgesics during the first week of treatment.

Results

One day following the intervention, there were no statistically significant differences between the two groups concerning pain levels, discomfort, and difficulties of mastication (P=0.293, P=0.166, P=0.538; respectively), but there was a statistically significant difference in the perceived swelling (P=0.012). On the seventh and 14th days of assessment, there were no statistically significant differences between the two groups regarding the previous variables. In the CORT group, the proportion of patients who were satisfied with treatment was approximately 94%, the proportion of those who found the treatment easy was 96%, 84% of patients wanted to repeat the procedure, and 92% of them would recommend this procedure to a friend.

Conclusions

There were no statistically significant differences in pain perception, discomfort, difficulties with mastication, and analgesic consumption between the interventional group and the control group. The perception of swelling was greater in the experiment group (the corticision group) at 24 hours following the first archwire engagement, and then it gradually decreased. Patients in both groups showed high levels of satisfaction following leveling and alignment. Those in the experimental groups showed a high level of willingness to undergo the same procedure again and a high level to recommend this procedure to a friend.

## Introduction

Pain is highly associated with orthodontic treatment as the main complaint [1]. The discomfort of harm or the possibility of harm and the sensory effect of this experience is a simple definition of pain [2]. The percentage of patients who underwent orthodontic treatment and suffered from different degrees of pain ranges between 65% and 95% [3]. Pain associated with orthodontic treatment affects daily life, such as difficulty in swallowing, speaking, and jaw movement limitation, and can lead to difficulty in mastication. All of these may lead to psychological discomfort from orthodontic treatment [4].

Fear of pain may prevent patients from undergoing orthodontic treatment, and painful experiences may lead to discontinuing their treatment [5]. Pain and discomfort have been evaluated during different orthodontic procedures from the first stages of elastomeric separators [6,7] through archwire replacements [8] and till the end of treatment and retainers wear [9]. The intensity of the pain associated with orthodontics increases with the increase in the duration of treatment [10].

With the advent of many surgically-based acceleration methods to shorten orthodontic treatment time, several studies have been published evaluating patient-reported outcomes such as pain, discomfort, functional impairments, satisfaction, and acceptability [11-15]. Corticision is defined as a surgical procedure did not associate with a flap lift (minimally invasive) [16-18]. The technical aspect of corticision was explained in some clinical case reports [16]. The latest randomized clinical trial RCT has shown that the corticision accelerates leveling and alignment in mild to moderate cases of crowding by 27% [17]. Regarding safety, a recent study has shown that the corticision is safe for root resorption and the formation of bone defects during decrowding [18].

Five trials in the literature evaluated the levels of pain associated with minimally invasive surgical procedures [11,19-22]. Split-mouth trial by Alfawal et al. compared pain and discomfort levels between laser-assisted flapless corticotomy and piezocision during canine retraction [11]. Their study showed significantly greater levels of pain in the surgical group at 24 hours following the surgical acceleratory intervention with a mean numeric rating scale (NRS) of 1.5. Alikhani et al. assessed those variables during canine retraction after applying micro-osteoperforations [19], pain levels were described as nearly similar with no significant differences between surgical and non-surgical groups at 24 hours postoperatively, while Gibreal et al. [12], Mehr et al. [21] and Charavet et al. [22] studied pain levels during the acceleration of piezocision assisted decrowding cases, and their patients had mild to moderate levels of pain and discomfort at 24 hours following piezocision.

Corticision as a minimally invasive surgical intervention has not been studied yet in terms of the associated pain and discomfort in comparison with ordinary treatment in patients with crowded lower teeth. According to the available literature, it seems that no study has yet evaluated the levels of pain, discomfort, acceptance, and satisfaction in patients treated orthodontically in conjunction with a corticision procedure.

## Materials and methods

Settings and study design

This study was conducted between March 2019 and August 2020 at the Department of Orthodontics, Faculty of Dentistry, Damascus University. This study was approved by the Research Ethics Committee of the Faculty of Dentistry at Damascus University (UDDS-249-2019HG/SRC-1827), registered at ClinicalTrails.gov (Identifier: NCT05250921), and the budget for postgraduate research at the Faculty of Dentistry at Damascus University has funded this research (Ref no: 83154207886DEN). This study was written according to the CONSORT statement [23]. This study was a randomized clinical trial with a two-arm parallel-group design.

Sample size assessment

G*power 3.1.7 software (Universität Düsseldorf, Düsseldorf, Germany) was used to estimate the sample size. The 25 mm pain level was accepted as the smallest clinically significant difference detected between the two groups on a visual analog scale (VAS). The standard deviation of this variable from a previous study was 23.75 mm [21]. Applying a paired t-test with a significance level of 5% and a power of 95%, 25 patients were required in each group. To compensate for any possible dropout, one patient was added to each group with a total sample size of 52 patients.

Participants and eligibility criteria

A clinical examination at Damascus University was conducted for 118 patients from the Archives of the Department of Orthodontics. Sixty-eight patients met the inclusion criteria. All patients' consents were obtained after the information sheets were distributed to them. Sixty-three of them agreed to join the current study. According to the a priori sample size calculation, 52 patients were randomly selected from the sampling frame. Then they were randomized into two groups with a one-to-one allocation ratio: the corticision group (CORT; n=26) and the control group (CONT; n= 26).

**Figure 1:**
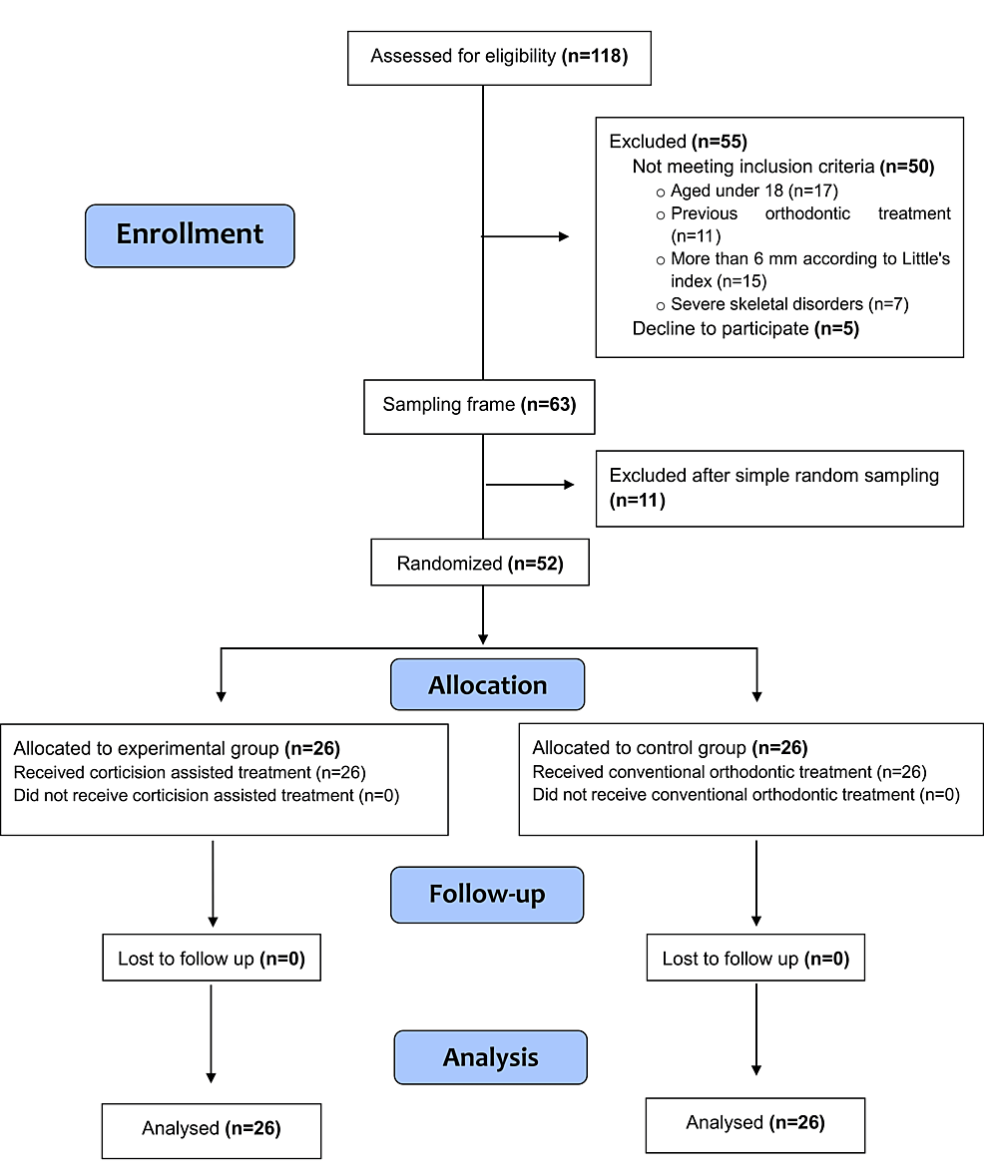
Consolidated Standards of Reporting Trials (CONSORT) flow diagram of patients' recruitment, follow-up, and entry into data analysis.

The inclusion criteria were: (1) age range between 18 and 24 years; (2) complete permanent dentition (except for the third molars); (3) mild to moderate crowding (2-6 mm according to Little's index); (4) absence of medications intake that would affect pain perception for at least one week before the beginning of the treatment. Exclusion criteria were: (1) systematic diseases that could affect bone and tooth movement and no contraindication avoid oral surgery; (2) medical conditions that affect tooth movement (e.g., Corticosteroid, [non-steroidal anti-inflammatory drugs] NSAIDs); (3) Previous orthodontic treatment; (4) poor oral hygiene or concurrent periodontal disease. The Consolidated Standards of Reporting Trials (CONSORT) flow diagram of patient recruitment, follow-up, and entry into data analysis is given in Figure 1.

Randomization, allocation concealment, and blinding

The randomization was performed using computer-generated random numbers with an allocation ratio of 1:1. The allocation sequence was hidden with numbered, opaque, and closed envelopes that were opened before treatment began. Corticision and initial wire have been applied by the corresponding author (MYH) without being blinded. Patients have been treated by the first author (MRS), who was aware to which each patient belonged. The blindness of participants was not applicable. Therefore, the blindness was applied only during data acquisition and data analysis (by one of the co-authors [ASB]).

First group: The corticision group

Corticision Procedure

**Figure 2:**
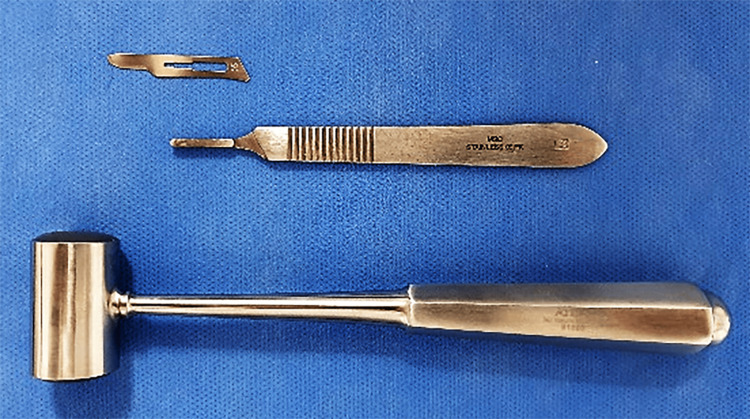
The surgical instruments used in the current trial: the surgical blade (No 15), the blade holder (No 3), and the surgical hammer.

**Figure 3:**
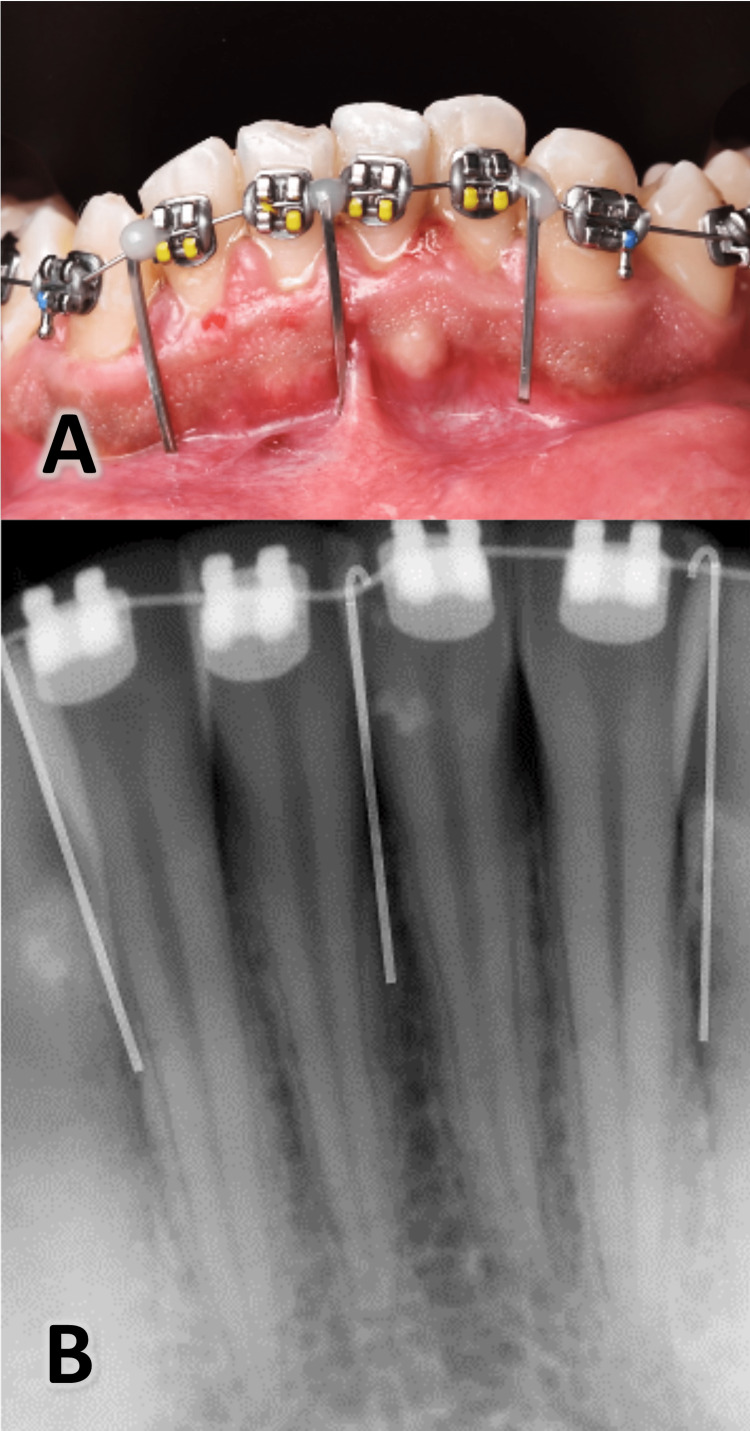
A: Three radiographic metal guides were used before taking digital radiographs. B: The appearance of radiographic metal guides in the taken image to help in determining the best places for the surgical intervention.

The fixed appliances were applied with MBT 022 prescription brackets (Pinnacle bracket system®, Ortho technology ™, Florida, USA). After bonding the brackets, patients rinsed their mouths with Chlorhexidine Gluconate 0.12% for one minute before the surgical intervention began. Then, a local infiltration anesthetic was applied to the anterior area of ​​the lower jaw (lidocaine hydrochloride 2% with epinephrine 1:80,000). Three incisions were applied with surgical blade No. 15 and hammer (Figure 2; Atlas Surgical Company, Delhi, India) between the roots of the central incisors and between the canine and the lateral incisor roots on each side of the lower jaw by relying on radiological guides made from 016 * 022 SS straight metal wires (American Orthodontics, Sheboygan, WI USA) to prevent damage to the roots near the incision’s sites (Figure 3). The length of the wires ranges from 10 to 15 mm, depending on the location of the mucogingival junction, and linked using flowable composite (Crystal‐Essence, Confidential, Louisville, CO, USA) with 0.14 stainless steel wire passing through the brackets neutrally. Periapical digital imaging for lower anterior crowded teeth (RVG 6100 sensor, Carestream, Kodak, USA) was performed.

**Figure 4:**
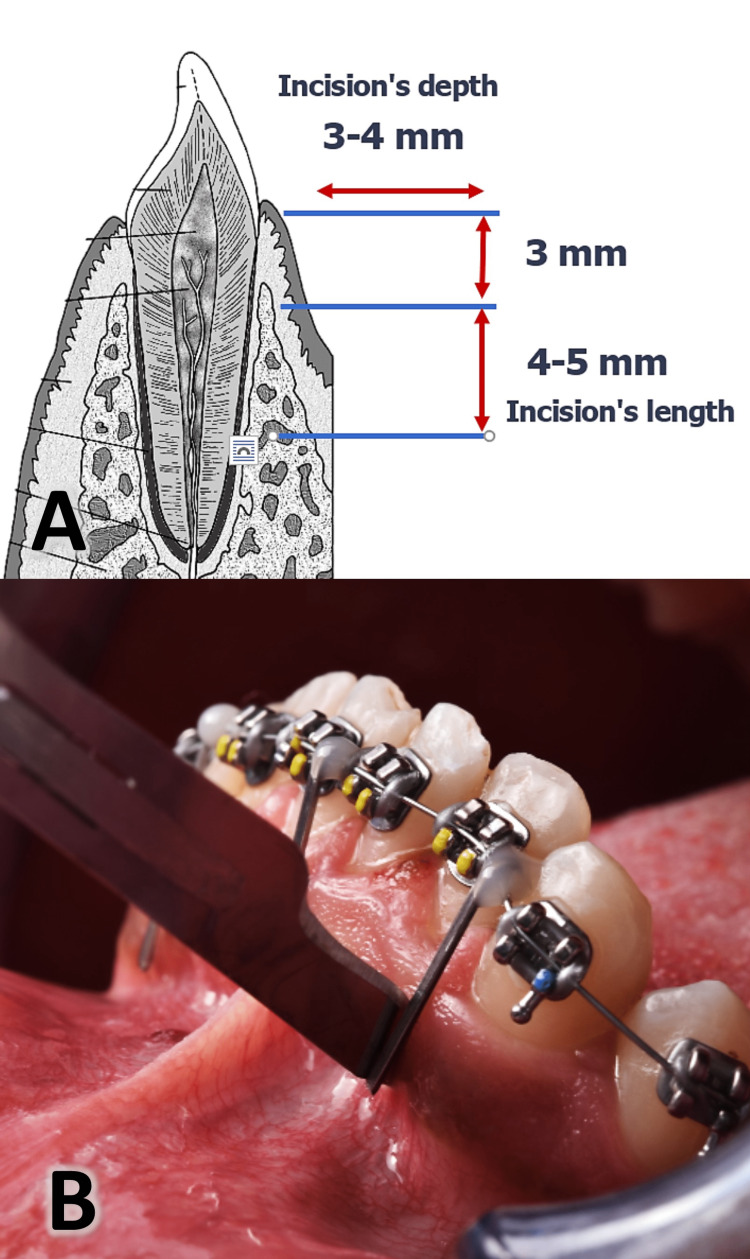
A: Three minimally invasive corticisions were performed using the surgical blade (no 15) with 4-5 mm long and 3-4 mm depth. B: The clinical appearance of the surgical blade showing the angle of penetration. The illustration in part A of this figure was drawn by the first author (MRS).

The incision's length was 4-5 mm, and the depth was 3-4 mm and 3 mm away from the gingival papilla (Figure 4). Corticision has been applied to each patient at the beginning of orthodontic treatment in three areas for one time before the first wire is applied. After the incisions are made and in the same session, 0.14-inch NiTi archwire wire has been applied (applying orthodontic force). Patients were instructed to avoid rinsing the mouth for 24 hours and to follow a soft diet after surgery. Patients were asked to start rinsing with 0.12% chlorhexidine solution thrice daily after 24 hours from surgery without any anti-inflammatories prescribed. The wounds were left without suturing to be healed by the second intention. In moderate/severe pain, patients were allowed to take Panadol® (acetaminophen; 500mg tablets) as long as the questionnaire was filled out firstly, recording the number of tablets taken and determining the day of taking it after the surgical procedure.

Orthodontic Procedures

The leveling and alignment stage was started with 0.014 Nitinol (NiTi) wire, then 0.16 * 0.16 NiTi wire, then 0.17 * 0.025 NiTi wire, and finally 0.19 * 0.25 stainless steel (SS) wire(American Orthodontics, Sheboygan, Wisc) was applied [24]. The leveling and alignment stage was finished when the last wire could be inserted neutrally in all slots of the brackets, and a little index was less than 1 mm [25].

Second group: The control group

Patients of this group underwent the same prescription brackets, archwire sequence, follow-up appointments (every 14 days), and archwires’ changing conditions.

Outcome measures

The degrees of receptivity, pain levels, and discomfort associated with corticision were evaluated for both groups using two questionnaires.

**Figure 5:**
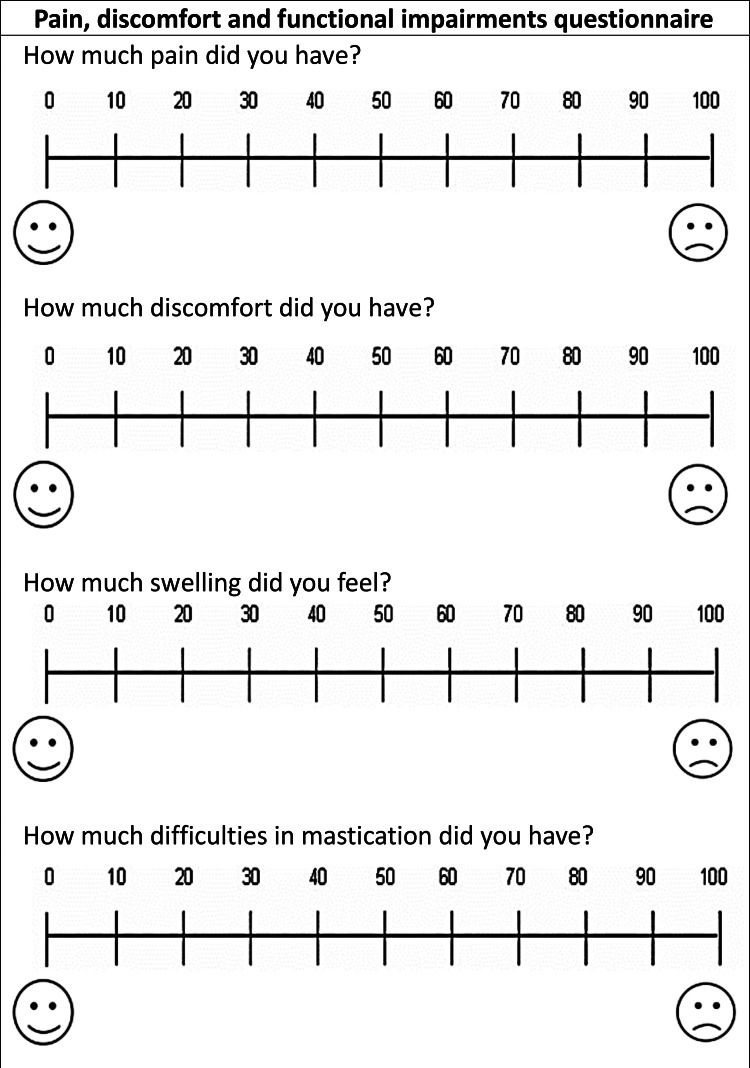
The first questionnaire was administered on the first day and 14 days following the onset of treatment for both two groups.

The first questionnaire was based on a set of questions - on the levels of pain, discomfort, and swelling, difficulties of mastication - that were answered by the Visual Analog Scale (VAS); it was given to patients at one day (T0), seven days (T1), 14 days (T2) following the onset of treatment (Figure 5). At the seventh day assessment (T1), a question was added about taking analgesics and the number of tablets used.

A line of 100 mm length was used, and the patient was asked to put a mark on the line that she/he feels reflects her/his current state, the scores of the scale were determined by measuring the distance in mm from the beginning to the point set by the patient (point (0): no pain, discomfort, swelling, difficulties of mastication, and point (100): the highest levels of pain, discomfort, swelling, difficulties of mastication that could be felt). The first archwire was applied to both groups, and the corticision was applied to the experimental group in the same period of the day [12 pm ± half an hour] to standardize the following assessment periods in all patients.

**Figure 6:**
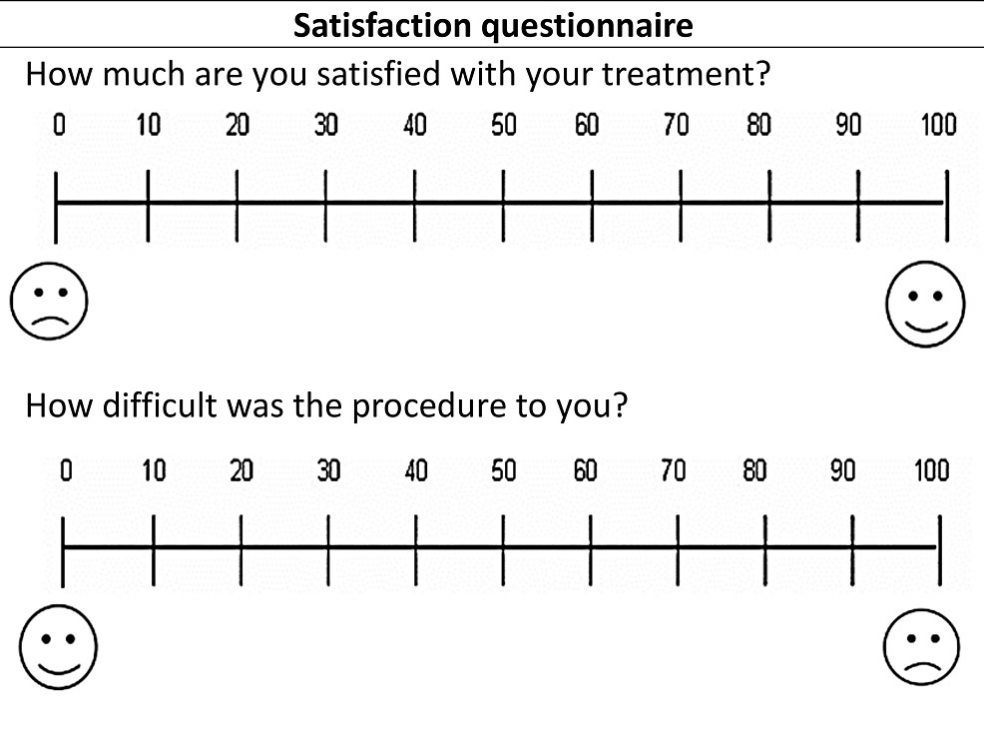
The second questionnaire was given at the end leveling and alignment stage in both groups.

The second questionnaire contains a set of questions for both groups - patients’ satisfaction with the procedure and ease of the procedure (Figure 6). In addition, two questions were directed specifically to the patients in the CORT group: the possibility of repeating the procedure and recommendation to a friend, which were answered using a two-point scale. This questionnaire was given to patients at the end of leveling and alignment stage. The questionnaires were numbered with serial numbers, and the distribution sequence was hidden from the researcher during the stage of extracting results to ensure the blinding of results.

Statistical analysis

Statistical analysis of the data was performed by one of the coauthors (ASB) using IBM Corp. Released 2011. IBM SPSS Statistics for Windows, Version 20.0. Armonk, NY: IBM Corp. Anderson-Darling tests were performed to determine the normality of the data distribution, and the distribution of the data was abnormal. Hence, nonparametric Mann-Whitney U tests were used to search for statistically significant differences between the two groups at each evaluation time. The Chi-Square test was used to study the significance of differences in taking analgesics between the two studied groups during the first week of treatment. The outcomes were assessed, and data were analyzed by one blinded assessor. For data analysis and evaluation of the measured outcome, it was single-blind. The results were considered significant if P ≤ 0.05.

## Results

Fifty-two patients were enrolled in this study, 26 in the CORT group (8 male/18) and 26 in the CONT group (6 male/20 female), patients mean age was 21.38 ± 1.05 years, and the basic characteristics of the sample are given in Table 1. The leveling and alignment time in the CORT group was 116.46 ± 15.97 versus 159.69±13.76 days in the CONT group (P-value <0.001). Hence, corticision-assisted treatment was 1.2 times faster than the traditional treatment, yielding a 27% reduction in treatment time.

**Table 1:** Basic sample characteristics N= Number of patients; SD: standard deviation; *Employing two-sample t-test; † Employing Chi-square test

Group	Gender N (%) male/female	P value†	Mean age in years (SD)		P-value^*^	Mean crowding in mm (SD)		P-value^*^
All sample (N=52)	14 (27%) / 38 (73%)	0.189	21.38	1.05	0.812	4.38	0.74	0.262
Corticision (N =26)	8 (31%) / 18 (69%)		21.46	1.76		4.57	0.75	
Control (N=26)	6 (23%) / 20 (77%)		21.30	1.49		4.20	0.87	

The statistical analysis ​​of the first questionnaire variables that was administered on the first, seventh, and 14^th^ day after the first archwire placement for both groups is given in Table 2.

**Table 2:** Descriptive statistics of patient-centered variables at one day, seventh day, and 14th day after first orthodontic archwire insertion in the two groups using visual analog scales. SD: Standard deviation, Min: minimum, Max: maximum, *Mann-Whitney U test

Variable	Group	Day 1			Day 7			Day 14		
		Mean	SD	Min-Max	Mean	SD	Min-Max	Mean	SD	Min-Max
Pain	Corticision	23.77	10.76	10-50	3.46	5.04	0-15	0.38	0.50	0-1
	Control	19.54	7.76	10-40	2.231	2.61	0-6	0.07	0.27	0-1
P-value		0.293*			0.738*			0.191*		
Discomfort	Corticision	21.69	10.70	10-50	4.00	3.48	0-10	0.38	0.65	0-2
	Control	17.69	9.53	10-40	2.30	2.62	0-6	0.15	0.37	0-1
P-value		0.166*			0.270*			0.488*		
Swelling	Corticision	23.23	9.57	10-40	4.46	4.14	0-11	0.38	1.38	0-5
	Control	12.08	10.12	0-30	2.30	2.92	0-10	0.38	1.38	0-5
P-value		0.012*			0.065*			1.000*		
Mastication	Corticision	27.46	14.75	5-50	1.30	2.09	0-5	0.15	0.37	0-1
	Control	22.62	14.15	0-40	0.61	1.55	0-5	0.38	0.76	0-2
P-value		0.538*			0.505*			0.681*		

On the first assessment day (T0), there were no statistically significant differences between the two groups for pain, discomfort, and difficulties of mastication (P=0.293, 0.166, 0.538, respectively). But there were statistical differences between the CORT and the CG only in the patient's feeling of swelling (P=0.012). The mean value of the feeling of swelling in the CORT group was 23.23, and in the CONT group was 12.08. After seven days of treatment (i.e., at T1), there were no statistically significant differences between the two groups for all variables (pain, discomfort, swelling, and difficulties of mastication; P=0.738, 0.270, 0.065, 0.505, respectively). Also, there were no statistically significant differences between the mean values of all variables at the third evaluation time (i.e., at T2). Thirty percent of the patients in the CORT group and 15% of the patients in the CONT group took medication for pain management (i.e., mean consumption of ≈ 346 mg and 115 mg, respectively) without any statistically significant differences between the two groups (P_taken-tables_=0.344, P_dose_=0.441; Table 3).

**Table 3:** The 7th-day assessment of the consumed analgesics in both groups N: Number of patients, SD: Standard deviation, Min: Minimum, Max: maximum, mg: milligram, *Mann-Whitney U test, † Chi-Square test

Questions		Day 7		P-value
		Corticision (N=26)	Control (N=26)	
Taken tablets	Yes: N (%)	8 (30.77%)	4 (15.38%)	0.344†
	No: N (%)	18 (69.23%)	22 (84.62%)	
Mean dose in mg (SD)		346.00 (555.00)	115.40 (299.60)	0.441*
Min-Max		0-1500	0-1000	

Subjects in both groups showed similar levels of ease and satisfaction with their treatment (P>0.05; Table 4).

**Table 4:** Descriptive statistics of variables measured in the second questionnaire in both groups at the end of the leveling and alignment stage N: Number of patients, SD: Standard deviation, Min: minimum, Max: maximum, *Mann-Whitney U test

Variable	Corticision (N=26)			Control (N=26)		
	Mean	SD	Min-Max	Mean	SD	Min-Max
Satisfaction with treatment	94.38	5.36	85-100	96.30	3.59	90-100
P-value	0.342*					
Ease of treatment	96.00	5.74	80-100	98.07	3.09	90-100
P-value	0.383*					

The Corticision group showed high levels of interest in undergoing treatment again and recommended their treatment to a friend (mean value ≈84%, and 92%, respectively; Table 5).

**Table 5:** Descriptive statistics of the patients' responses at the end of the leveling and alignment stage (in the corticision group) regarding their willingness to undergo this procedure again and the possibility to recommend this procedure to a friend. N= Number of patients

Questions	Only the corticision group (26 patients)	
	Answered 'Yes' N (%)	Answered 'No' N (%)
Willingness to repeat this procedure again	22 (%84.62)	4 (%15.38)
Recommendation to a friend	24 (%92.31)	2 (%7.69)

Harms

Patients did not suffer from complications during the entire duration of the study, such as dizziness, lower lip numbness, hematomas, scars, or any other short-term post-surgical side effects.

## Discussion

This study is the first RCT to assess levels of pain and discomfort between corticision and traditional orthodontic treatment in the leveling of mild and moderate crowded lower anterior teeth with the non-extraction method. The VAS instrument was used to assess pain perception due to its accuracy compared to other measures [22,26]. Questionnaires began to be filled 24 hours after the first archwire placement to avoid the analgesic effect of the local anesthetic used for the CORTgroup.

Pain perception

Regarding pain perception of the CORT, pain peaked 24 hours after the first archwire placement (mean value =23.77 mm), then- on the seventh day -it decreased to (3.46mm), and pain levels were close to zero on the 14^th^ day after starting the treatment. In all the three times measured (T0, T1, T2), there were no statistically significant differences between the two groups.

Four trials studied pain levels accompanying the acceleration of leveling and alignment with minimally invasive surgical methods [12,21,22,27]. For treatment methods, three studies were carried out with the non-extraction method [21,22,27] and one with the extraction method. For the location of the intervention, two studies were accomplished by intervention on the lower jaw only [12,21], and two studies on both jaws [22,27]. Three of these studies were done without radiological guides [21,22,27] and one with them [25]. All these studies were executed by using the piezocision technique.

In Charavet et al. study [22], 22 incisions were applied between teeth roots (5 mm length and 3 mm depth for each incision). Yavuz et al. study [27] applied 22 incisions between teeth roots (7 mm length and 3 mm depth for each incision). While Mehr et al. study [21] applied three anterior incisions (4 mm length and 1 mm depth for each incision). And in Gibreal et al. study [12], five anterior incisions were applied (5-8 mm length and 3 mm depth for each incision). In all these trials, there were no statistically significant differences between the studied groups (experimental and control groups) in all assessment times in relation to pain levels.

When evaluating post-intervention pain at the same assessment time in the current trial and the aforementioned studies (i.e., at 24h postintervention), the experimental subjects’ levels of pain were greater than that reported in the RCTs of Charavet et al., Yavuz et al., Mehr and Gibreal et al. in comparison with the current trial (i.e., mean values: 60 mm, 30 mm, 30.28 mm, 32.06 mm, 23.77 mm, respectively). These results could be explained by the fact that corticision was not associated with flap lifting, the number of incisions was minimal (three only), and the dimensions of the intervention were precise (4-5 mm in length and 3-4 mm in depth). The safety of the roots and their periodontal ligaments were taken into consideration by using radiological guides, and this reduced the pain after surgery.

Discomfort, swelling, and the difficulties of mastication

Regarding discomfort, swelling, and the difficulties of mastication for all assessment times, there were no statistically significant differences between the two study groups except the swelling values ​​of CORT 24 hours after the first archwire placement, which was greater than the value ​​of the control group by a statistically significant difference (mean value of CORT in =23.23mm, CONT=12.08mm). However, it did not exceed the presumed threshold of 20 mm difference on the VAS scale and therefore is not considered clinically significant. And it decreased significantly after seven days and 14 days (P=0.045, P≈1, respectively). This could be explained by the intervention with the surgical blade and hammer causing precise, controlled incisions without any harmful effect.

These variables (discomfort, swelling, and the difficulties of mastication) were studied in two studies to accelerate tooth movement with minimally invasive surgical methods [12,28]. The study of Al-Kebsi et al. [28] for accelerating canine retraction by MOPs found that the values ​​of swelling after the first day of the beginning of canine retraction were greater in the experimental group (P=0.05). This was consistent with the Gibreal et al. [12] study to accelerate the leveling and alignment by piezocision (P=0.011). This was also consistent with the results of the current study.

In both of these studies, there were no statistically significant differences for all variables studied and for all evaluation times. But the comparison with the study of Alkebsi et al. may not be accurate because of differences in the type of tooth movement and its biomechanics, the type of force applied, and the method of acceleration.

Analgesic medications taken

Gibreal et al., Charavet et al., and Mehr et al. studies [12,21,22] included analgesic medications taken during accelerated orthodontic treatment. The Gibreal et al. study [12] found that the patients did not take any analgesic drug, while in the Mehr et al. study [21], 71% of the experimental group and 66% of the control group took analgesics

And with Charavet et al. study [22], the rate of analgesic taking was 1.2 g for the control group and 2.2 g for the experimental group. Whereas in the current study, the percentage of patients taking analgesics and the average drug dose were mentioned, 30% of the CORT patients took ≈ 346 mg of painkillers, and 15% of the CONT patients took ≈115 mg of painkillers during the first week of treatment, without any statistical differences between the two groups. This means that the corticision did not adversely affect the perception of pain.

Satisfaction with the procedure

The RCTs of Gibreal et al., Charavet et al., and Mehr [12,21,22] included levels of satisfaction, the possibility of repeat treatment, and recommending a friend. Satisfaction levels in these studies were greater in the experimental groups, and the patients showed the ability to repeat treatment and the desire to recommend a friend, and this is consistent with the results of the current study, in which 94% of patients in the corticision group were satisfied with procedures as well as most of them were ready to repeat it and would recommend to a friend.

Ease of the procedure

Regarding the ease of the procedure, it was proposed with Mehr et al. study of piezocision [21] and showed that 84%-86% of patients consider piezocision an easy procedure. This was, in principle, consistent with the current study applied to corticision, which showed that 96% of patients consider corticision an easy procedure. This could be explained by the application of flapless corticision was rapid, painless, with a minimum of incisions (only three incisions), and without sutures.

Limitations

The current study had some limitations First, it was necessary to assess the state of periodontal tissue, gingival recessions, teeth vitality, and long-term complications. Secondly, there was a need to assess root resorption and development of dehiscence and fenestration after corticision. Finally, it was necessary to evaluate corticision with other noninvasive methods and with different orthodontic movements.

## Conclusions

There were no statistically significant differences in pain perception, discomfort, difficulties with mastication, and analgesic consumption between the interventional group and the control group. The perception of swelling was greater in the experiment group (the corticision group) at 24 hours following the first archwire engagement, and then it gradually decreased. Patients in both groups showed high levels of satisfaction following leveling and alignment. Those in the experimental groups showed a high level of willingness to undergo the same procedure again and a high percentage to recommend this procedure to a friend.
